# International Conference on Advances in Radiation Oncology (ICARO): Outcomes of an IAEA Meeting

**DOI:** 10.1186/1748-717X-6-11

**Published:** 2011-02-04

**Authors:** Eeva K Salminen, Krystyna Kiel, Geoffrey S Ibbott, Michael C Joiner, Eduardo Rosenblatt, Eduardo Zubizarreta, Jan Wondergem, Ahmed Meghzifene

**Affiliations:** 1 STUK, Finnish Radiation and Nuclear Safety Authority and Dept. of Radiation Oncology Turku University Hospital, Finland; 2 Department of Nuclear Sciences and Applications, Division of Human Health, International Atomic Energy Agency, P.O. Box 100, Vienna, Austria; 3 Department of Radiation Oncology, Northwestern University, 1653 W. Congress Pkwy, Chicago, IL 60612, USA; 4 Radiological Physics Center, UT M.D. Anderson Cancer Center, Box 547, 1515 Holcombe Blvd Houston, TX 77030, USA; 5 Dept. of Radiation Oncology, Wayne State University School of Medicine, Gershenson Radiation Oncology Center, 4100 John R. Detroit, MI 48201-2013

## Abstract

The IAEA held the International Conference on Advances in Radiation Oncology (ICARO) in Vienna on 27-29 April 2009. The Conference dealt with the issues and requirements posed by the transition from conventional radiotherapy to advanced modern technologies, including staffing, training, treatment planning and delivery, quality assurance (QA) and the optimal use of available resources. The current role of advanced technologies (defined as 3-dimensional and/or image guided treatment with photons or particles) in current clinical practice and future scenarios were discussed.

ICARO was organized by the IAEA at the request of the Member States and co-sponsored and supported by other international organizations to assess advances in technologies in radiation oncology in the face of economic challenges that most countries confront. Participants submitted research contributions, which were reviewed by a scientific committee and presented via 46 lectures and 103 posters. There were 327 participants from 70 Member States as well as participants from industry and government. The ICARO meeting provided an independent forum for the interaction of participants from developed and developing countries on current and developing issues related to radiation oncology.

## Introduction

### ICARO: Advancing Radiation Oncology

All countries are facing an increased demand for health services. In cancer care, there are more expensive demands in diagnosis and treatment, including radiation therapy, and systemic therapies. Radiation therapy is a cost-effective method of treating cancer, yet it is unavailable in many low income countries throughout the world. In high income countries, the ratio of treatment machines to population may be as high as six per million individuals, but in many low and middle income (LMI) countries, the ratio may be as low as one per 10-70 million individuals. Twenty IAEA Member States have no radiotherapy services at all and many low-income countries have only basic equipment and often few trained and qualified staff, for which there is a global shortage.

The ICARO meeting provided an overview of topics and issues facing the modern radiation oncologist with an emphasis on advanced technologies and covering topics as shown in Table 1. Invited speakers were prominent in the field, many with experience in LMI countries. Parallel sessions were held on topics specific for a subset of the audience (medical physicists and radiation oncologists) along with side events to discuss very specific issues such as QA in clinical trials and collaboration with commercial companies. Summaries of individual sessions are highlighted in the text.

**Table 1:** Overview of ICARO programme topics

Main topic	Advanced techniques (*) in teletherapy
Clinical sessions/clinical practice	Advances in chemo-radiotherapy in cervical and head-and-neck cancer Current trends in brachytherapy Radiotherapy in paediatric oncology Reducing late toxicities Altered fractionation
Training sessions/educational	How to set up a QA programme? Commissioning and implementing a QA programme for new technologies Transition from 2D to 3 D CRT and IMRT Training, education and staffing: evolving needs/getting ready to transition to the new technologies Cost and economic analysis in radiation oncology

Planning new activities	PACT meeting with manufacturers of diagnostics and radiotherapy equipment Global quality improvement for clinical trials in radiation oncology

Controversial topics and debates	Co-60 - no time for retirement? IMRT-are you ready for it? Do we need proton therapy?

(*) For the purposes of this report, "advanced technologies" include 3-D conformal radiation therapy (3D-CRT), intensity modulated radiation therapy (IMRT), image-guided radiation therapy (IGRT), adaptive radiation therapy (ART), respiratory-gated radiation therapy (RGRT), particle radiation therapy, and image-guided brachytherapy (IGBT) in all aspects; planning, treatment delivery, and quality assurance.

Conclusions based on interaction and discussion between participants focused on inadequacies of current systems:

• There are many low income countries with no or very basic diagnostic and treatment facilities.

• Low and middle income (LMI) countries have an increasing number of cancer patients who present with advanced stage disease, with few radiotherapy facilities. Palliative treatment is common, but there are an increasing number of potentially curable patients.

• Demand for radiotherapy services in LMI countries will increase dramatically over the next 20 years.

### Diagnostic Imaging Requirements

Many successes in the treatment of cancer with radiation therapy are related to earlier diagnosis, a multidisciplinary approach to cancer diagnosis and treatment, and more precise delivery of radiation therapy. Recent advances in radiation therapy planning and delivery allow improved normal tissue sparing and escalation of the tumour dose compared to conventional techniques (2D RT). These improvements require precise definition of the tumour target, especially when three-dimensional conformal radiation therapy (3D-CRT) and intensity-modulated radiation therapy (IMRT) are under consideration. Often this requires the use of dedicated computed tomography (CT) scanning, which can be integrated into treatment planning software. X ray exposure associated with extra imaging must be considered. There is a general increase of diagnostic X ray exposure worldwide in health care. The risks of radiation exposure in radiation treatment planning may be mitigated by requirements for precise treatment delivery, and developments in CT equipment may help reduce this exposure.

### Current role of cobalt-60

A debate was held regarding the utility of cobalt-60 teletherapy in routine practice. Cobalt-60 units have traditionally been "friendlier" treatment machines to place in new low-resource departments with regards to cost, the training required, treatment delivery, planning, and maintenance [1,2]. However, the production cost of cobalt-60 sources is increasing and there are heightened security concerns. Modern sophisticated cobalt machines are more costly, reflecting increasing pricing. At the same time, there has been a relative decrease in the cost of small, single-energy linear accelerators (linacs), making the two modalities roughly comparable when combining initial and ongoing costs. Cobalt-60 sources must be replaced every 5-6 years, requiring disposal of the old sources (an increasingly costly and logistically difficult problem) and this expense must be weighed against cost, commissioning, training, and maintenance of a linac which has a useful lifespan of 10-12 years. QA programmes are more complex for linac units. In some LMI countries, the frequent lack of stable electrical power can interfere with the smooth operation of linacs. Service personnel may have to travel long distances, and parts may not be readily available. Frustrations were expressed with expensive and delicate equipment that was rendered unusable by simple problems, especially when requirements for infrastructure, staff training and maintenance were not initially recognized.

The current and emerging need for teletherapy units in developing countries cannot be met by cobalt machines alone. Selecting the right equipment should be mainly based on local radiotherapy experience and case-mix, as well as on financial, technical and human resources available. Many LMI countries may benefit from the use of both cobalt units and linacs with use based on complexity of treatment.

Conclusion:

• There remains a role for cobalt teletherapy in LMI countries. New technical developments may allow the introduction of highly-conformal treatment techniques with cobalt but this increases the cost to the level of medical linear accelerators.

### Implementation of advanced technologies

A series of keynote lectures discussed the underlying hypothesis for the use of advanced technologies in radiation therapy, discussing the assumption that improved dose distribution leads to improvement in clinical outcomes.

New treatment technologies are evolving at a rate unprecedented in radiation therapy, paralleled by improvements in computer hardware and software. The challenging use of highly precise collimators in the IMRT setting, small fields, robotics, stereotactic delivery, volumetric arc therapy and image guidance has brought new challenges for commissioning and QA. Existing QA guidelines are often inadequate for some of these technologies. New QA procedures are needed and are under development. In the meantime, the existing paradigm of commissioning followed by frequent QA should continue, with attention paid to the capabilities offered by the new technologies. Risk management tools should be adapted from other industries to help focus QA procedures on where they can be most effective.

These techniques allow assessment of changes in the tumour volume and its location during the course of therapy (*interfraction motion*) so that re-planning can adjust for such changes in an adaptive radiotherapy process. Some target volumes move during treatment due to respiration (*intrafraction motion*), especially those in the lung, liver and pancreas. Advanced techniques for compensating for such motion are already commercially available and include respiratory gating, active breathing control and target tracking.

The speakers advised to approach the implementation of the new technologies with caution. If the identification of target tissues is uncertain when margins around target volumes are tight, the likelihood of geographic misses or under-dosing of the target increases. Movement of the target with respiration or for any reason during treatment increases the risk of missing or under-dosing the target. Since in some instances IMRT uses more treatment fields from different directions, its use may increase the volume of normal tissue receiving low doses which might lead to a higher risk of secondary cancers. With the introduction of any advanced technology, such as IMRT and IGRT, data should be collected prospectively, to allow a thorough evaluation of cost-effectiveness and cost-benefit [3,4].

A debate on ***IMRT: Are you ready for it? ***brought together panel members who represented various views from all regions of the world, including high and LMI countries. A modality such as IMRT offers the theoretical potential to increase radiation dose to tumour target volumes while sparing normal tissues. Health economics was identified as a key motivator in the adoption of IMRT. There is still a lack of randomized trials supporting robust evidence of clinical benefit of IMRT in many tumour sites. There is little prospective data demonstrating that IMRT provides clinical benefit other than improved dose distribution [5]. Unexpected toxicities and recurrences have been reported in the literature [3].

In the USA, where such trials could be done, there is great difficulty recruiting patients to the non-IMRT arm because hospitals promote IMRT in order to stay economically competitive. In Europe, IMRT is used somewhat less, with figures for Belgium being approximately 50% and the UK less than 50%. In India and South Africa, the figure drops to 25%. Comparative case series [6,7] and some phase-III trials [8,9] have been completed in the USA, Europe and Asia. The overall conclusion from these trials is that there is evidence of reduced toxicity for various tumour sites by the use of IMRT. The evidence regarding local control and overall survival is generally inconclusive [5].

Advanced technologies of radiation treatment such as IMRT require optimal immobilization and image guidance techniques. There was debate as to whether image guidance was always required with IMRT to ensure accurate delivery. Whether image guidance was necessary daily was also debated and this may be necessary in specific cases, such as when immobilization is not optimal or when hypofractionation is used. Other techniques to control organ motion during treatment such as respiratory-gating and breath-hold techniques may be necessary when reduced target volumes are considered.

A survey on IMRT conducted in the USA [10] determined that the three main motivators for implementing this modality were normal tissue sparing (88%), allowing dose-escalation (85%) and economic competition (the desire to remain competitive) (62%). In addition, 91% of non-users planned to adopt IMRT in the future.

Image Guided Radiation Therapy (IGRT) can be defined as increasing the radiotherapy precision, by frequent imaging the target and/or healthy tissues just before treatment and acting on these images to adapt the treatment [11]. There are several image-guidance options available: non-integrated CT scan, integrated x-ray (kv) imaging, active implanted markers, ultrasound, single-slice CT, conventional CT or integrated cone-beam CT.

A survey on IGRT in the USA [12] revealed that the proportion of radiation oncologist self-declared users of IGRT was 93.5%. However, when the use of megavoltage (MV) portal imaging was excluded from the definition of IGRT, the proportion using IGRT was 82.3%. Among IGRT users, the most common disease sites treated are genitourinary (91.1%), head and neck (74.2%), central nervous system (71.9%), and lung (66.9%).

Conclusions:

• Robust clinical trials are necessary to demonstrate the benefits of advanced technologies before they are adopted into widespread use.

• A new and unproven technology should not be universally adopted as a replacement for established proven technologies.

• LMI countries should avoid the risk that by hasty implementation of new technologies, patients would no longer have access to established methods of treatment.

### Introduction of advanced technologies: the radiation oncologist perspective

It was noted that the implementation of advanced radiotherapy technologies tends to distance the physician from the patient, a trend that needs to be consciously counterbalanced by a more personal and holistic approach. In addition, it makes it more and more difficult to intuitively understand the relationship between the radiation fields and the patient's anatomy. Whereas with 3D conformal radiation therapy, the physician can rely on port films to assess the irradiated volume, with IMRT the physician must rely on tools such as computer simulations and dose-volume histograms (DVH). Users of advanced technologies should be cautioned not to allow themselves to become too dependent upon the technology itself. It was also recommended that advanced technologies such as IMRT and IGRT should not be acquired until physicians and hospital staff are fully experienced with advanced treatment planning techniques in 3D conformal therapy.

Modern 3D approaches including IMRT introduce new requirements in terms of understanding of axial imaging and tumour/organs delineation. Recent literature points to an uncertainty level at this stage known as "inter-observer variations". Efforts continue to harmonize the criteria with which tumours, organs and anatomical structures are contoured and how volumes are defined.

### Introduction of advanced technologies: the medical physics perspective

The introduction of IMRT and stereotactic radiation therapy procedures brings special physics problems. For example, it is required that calibrations be performed in small fields, for which the dosimetry is challenging, and no harmonized dosimetry protocol exists. Use of the correct type of dosimeter is critical, and errors in measurement can be substantial. Several new treatment machines provide radiation beams that do not comply with the reference field dimensions given in existing dosimetry protocols complicating the accurate determination of dose for small and non-standard beams.

The introduction of highly precise collimators in the IMRT setting, small fields, robotics, stereotactic delivery, volumetric arc therapy and image guidance has brought new challenges for commissioning and QA. The existing QA guidelines are often inadequate for the use of some of these technologies. New QA procedures are needed and are under development. In the meantime, the existing paradigm of commissioning followed by frequent QA should continue, with attention paid to the capabilities offered by the new technologies. Risk management tools should be adapted from other industries, to help focus QA procedures on where they can be most effective [13].

It was observed by several speakers that IMRT requires increased attention to physics and dosimetry, more equipment, training and technical support, and more time for quality assurance. Specific issues mentioned included the critical need for accurate calibration of the position of multi-leaf collimator leaves, and the precise modelling of radiation dose distributions especially in the penumbra region produced by MLC leaves. The veracity of data transfer from the treatment plan to the treatment machine is critical whether it be by electronic or manual means, and should be included in QA programmes.

### Fractionation

Advanced technologies provide an opportunity for the acceleration of treatment without excessive risk to normal tissue [3]. Hypofractionated treatments are more convenient to patients and caregivers. But convenience is not enough to make hypofractionation a mainstay treatment. Much of this subject is still surrounded by ongoing controversy. The avoidance of dreaded late effects of hypofractionation obviously cannot be confirmed without long and careful follow-up [14].

In curative and palliative treatment, several trials of hypofractionation in common cancers have shown comparable clinical outcomes to conventional fractionation. These schedules vary for different diseases with fractions >2 Gy given daily to once weekly. Common cancers, such as breast cancers, can be successfully treated in three weeks rather than in five weeks [15]. Advanced technology radiation therapy (3D CRT and IMRT) may provide an opportunity for the study of tissue tolerance as high doses per fraction can be delivered to small tumour volumes while normal tissues receive conventional fractionated radiation.

Investigators treating common diseases such as prostate and breast cancer are using non-ablative hypofractionation in patients with curable tumours. This strategy tends to be well received in environments where the cost-savings associated with fewer fractions is important. In some cases, such hypofractionation has a biological rationale for improving the therapeutic ratio [14].

Conclusions:

• There is significant published experience with the use of hypofractionated regimens in breast, [15,16] prostate [17,18] brain/body [19] and palliative radiotherapy.

• The use of hypofractionated regimens can be particularly useful in limited-resource centres overloaded with large number of patients.

### Current role of proton therapy

The dosimetric advantage of charged-particle beam radiotherapy derived from the Bragg peak was emphasized. Protons and other particles have been used for decades for ocular melanomas, base of skull tumours, and brain tumours where radiation dose escalation using photons was not possible due to normal tissue constraints. The first hospital-based proton facility was opened in Loma Linda (USA) in 1999 [20]. Since then, over 30 particle-based facilities have opened and another 30 are in the planning stages worldwide, primarily for the treatment of cancer patients. Until recently, the significant capital expenditure required for the establishment of a proton facility has limited the availability of this form of radiation therapy in many areas of the world. This modality is expensive, time consuming, and requires special expertise. The cost of treatment is significantly higher than conventional 3D-CRT.

During the ICARO meeting, a debate addressed the question: *Is there a need for proton therapy? *Proponents and opponents considered the following three propositions: (1) Proton dose distributions with currently available equipment are likely to be of real benefit to patients; (2) On the basis of clinical evidence, protons should be made available for radical radiotherapy to many more patients; and (3) Further technological developments will make proton therapy more cost effective.

The speakers described the advantages offered by proton beams, such as increased conformality of dose distributions to target volumes and lower doses to non-target tissues. The speakers provided examples of exquisitely-shaped dose distributions that can be achieved with both photon IMRT and with spot-scanned protons. It was mentioned that the improved dose distributions with protons might offer significant benefits to paediatric patients, although the benefits might require some years to become detectable and may not yet be readily measureable. No benefit has been demonstrated in the treatment of prostate cancer, including following completion of one randomized trial [21] although proton therapy appears at least to match the high success rates and low toxicity available with photon IMRT [22,23]. Future advances in proton therapy equipment and technologies are expected to provide even greater benefits through improved dose distributions and patient throughput, but challenges in standardizing calibrations, treatment parameters, and the relative biological effectiveness must be addressed first. Proton treatment of cancer patients should be done preferably within clinical studies for collecting data, which allows clear comparison with conventional photon treatment, thereby defining the role of proton therapy precisely within radiation oncology. Reported biochemical disease-free survival rates after carbon ion radiotherapy appear higher than with modern photon IMRT and proton RT especially for patients with high-risk prostate cancer [24].

Slater and co-workers [23] report a 5-year NED rate of 57% while a 5-year NED rate of 51% was reported for conventional RT with photons [25].

Photon IMRT yields a biochemical DFS rate of 81% at 3 years, whereas severe toxicity rates to the genitourinary system and the rectum are higher as compared with the rates reported by Akakura and co-workers with carbon ions (10% vs. 1.4%) [24].

Conclusions:

• Physical dose distributions of proton beams are superior to those of photons

• The cost of establishing and maintaining proton facilities is significant

• Clinical trials are underway and over the next several years an increased amount of clinical data will become available

• The question of whether the clinical gains from proton therapy will outweigh the costs is an unresolved issue.

### Brachytherapy

The session on brachytherapy highlighted recent advances in this modality of radiation therapy. In the past, brachytherapy was carried out mostly with Radium (^226^Ra) sources. Currently, use of artificially produced radionuclides such as ^137^Cs, ^192^Ir, ^60^Co, ^198^Au, ^125^I, and ^103^Pd has rapidly increased.

Brachytherapy is an essential component of the curative treatment of cervical cancer (a very common disease in many LMI countries) and cannot be replaced by other modalities in this setting. High dose-rate (HDR) brachytherapy is preferable to low dose-rate (LDR) for departments with limited resources that treat a large number of patients with cervical cancer. New systems using a miniaturised ^60^Co source are becoming very popular [26-29]. This is due to the fact that ^60^Co based HDR systems require source replacement approximately every 5 years while ^192^Ir requires replacement every 3-4 months. This represents a significant advantage in terms of resource sparing, import of radioactive sources into countries, regulatory requirements and additional workload [30].

Over the last decade developments in imaging, computer processing and brachytherapy systems and applicators have made possible to implement three-dimensional treatment planning based on cross sectional imaging with the applicators in place using CT or MRI. This has been successfully developed for the brachytherapy of cervical cancer [31-33].

Individual departments in low-middle income countries should carefully weight the advantages and disadvantages of adopting this system which implies expenses in terms of applicators and requires readily available MRI services dedicated to the brachytherapy unit or department.

In prostate cancer, excellent long-term tumour control can be achieved with brachytherapy, and this approach is considered a standard treatment intervention associated with comparable outcomes to prostatectomy and external beam radiotherapy for patients with clinically localized disease [34]. In low-risk disease patients, seed implantation alone (monotherapy) achieves high rates of biochemical tumour control and cause-specific survival outcomes. For those with intermediate risk and selected high-risk disease, a combination of brachytherapy and external beam radiotherapy is commonly used.

In the treatment of prostate cancer, the radioactive sources can be implanted permanently using ^125^I seeds [35] or as a fractionated temporary implant using a high dose-rate stepping source. Although the experience with seed implantation is more extensive and the results mature [36], the use of HDR brachytherapy as monotherapy or combined with external beam therapy is becoming more popular in radiotherapy departments that already have a HDR brachytherapy device, thus avoiding the costs and procedures of importing ^125^I seeds for each individual patient [37,38]. HDR brachytherapy offers several potential advantages over other techniques. Taking advantage of an afterloading approach, the radiation oncologist and physicist can more easily optimize the delivery of radiation therapy to the prostate and reduce the potential for under-dosage ("cold spots"). Further, this technique reduces radiation exposure to the care providers compared to permanent seed implantation. Current approaches are employing HDR monotherapy for intermediate risk patients avoiding the need for supplemental external beam radiotherapy [39].

Both approaches are time/effort consuming and require careful attention to technical detail. An imaging method (commonly trans-rectal ultrasound) has to be used during seed or needle implantation. The procedures require attention to accurate dosimetry and normally there is a "learning curve" for the whole brachytherapy team.

The introduction of HDR brachytherapy as a treatment modality carries with it additional concerns related to QA and radiation protection. The very principle of HDR brachytherapy is based on working with a very high activity radiation source, and short treatment times. Therefore, all centres implementing HDR brachytherapy must establish a written policy on QA and pay utmost attention to basic principles of radiation protection.

HDR treatments dramatically increase the physician and physicist resources that must be allocated to brachytherapy while reducing the needs for inpatient hospital beds. The relative cost and availability of these resources should be compared, and the cost-savings, compared with the cost of amortizing the capital investment required and the cost of source replacement and machine maintenance [40].

### Education and training

An important theme echoed by several speakers and the audience was the global shortage of skilled professionals. It was noted that while short-term and local solutions have been devised, there was a need for a long-term strategy to produce trainers and educators who could increase the supply of adequately trained staff. Training must be adapted to both the working environment and the level of complexity of the available technology; little benefit is derived by a trainee or the trainee's institution when the education addresses a technology not available in his or her own country.

There is clearly a role for networking on the national and regional levels to support education networks. The role of the IAEA in education and training through national and regional training courses and development of teaching materials and syllabi was recognized.

Conclusions:

• There is a worldwide shortage of qualified radiotherapy professionals

• Specialized education and training must be provided to meet this demand.

### Cost considerations

In the delivery of routine radiotherapy, most expenditure is in personnel costs, followed by equipment costs and depreciation. Each institution has its own requirements for equipment and personnel. These requirements are based on the type and stages of encountered cancers ("case-mix"), the type of equipment and facilities availability, local work practices, and method of financing, maintenance costs, and down-time and life cycle of treatment machines. Many countries have observed the cost of radiation therapy delivery to have increased annually.

The IAEA has developed a cost estimator [41] which takes into account potential workload based on cancer incidence and staging, overhead and indigenous costs of personnel and facilities, in addition to equipment costs. The costs of a cobalt-60 machine when including ultimate source disposal, has become similar to a low energy linear accelerator, but training, personnel, and maintenance costs are lower and reliability is higher.

Cost-effectiveness analysis (CEA) is a form of economic analysis that compares the relative costs and outcomes (effects) of two or more courses of action [42]. Cost-effectiveness analysis is distinct from cost-benefit analysis, which assigns a monetary value to the measure of effect [43]. Cost-effectiveness analysis is often used in the field of health services, where it may be inappropriate to monetize health effect. Typically, CEA is expressed in terms of a ratio where the denominator is a gain in health from a measure (years of life) and the numerator is the cost associated with the health gain. The most commonly used outcome measure is quality-adjusted life years (QALYs) [44].

Cost effectiveness can be measured in gain in quality adjusted life years (QALY), cost per QUALYs, cost per year of life gained or cost per loco-regional failure avoided.

When assessing the usefulness of newer advanced technologies, cost effectiveness can be measured several ways:

Is the number of patients to whom services are delivered increased? (Improved access). Are cure-rates increased? (improved curability). Is toxicity significantly reduced? (Improved therapeutic index) What is the ultimate objective for the introduction of a new technology? And what are its cost implications?

Systematic studies of the newer technologies seem required following the methodologies of health technology assessment and the dissemination of the results in a form that is accessible to clinicians, mangers and the public. Unfortunately, much of the evidence indicates that it is difficult to influence practitioners simply by producing and disseminating information.

Although extremely important, education and training costs are not usually considered in these formulas. Cost effectiveness can often be improved by optimal use of conventional technologies and better work practices. For instance, hypofractionation can increase patient throughput while maintaining the same outcome in selected indications.

Radiotherapy services in LMI countries need high level government commitment to mobilize the necessary funds of approximately $5-6 million necessary to establish a basic cancer centre. Such projects, when completed, take at least 5 years to make a noticeable difference in the health care system as a whole.

Conclusions:

• ICARO speakers and panellists emphasized that each country should have a comprehensive plan for cancer control.

• The value of advanced technology must be assessed relative to the indigenous needs and structures of the country. It is important that radiation oncology be part of health planning for a country/community, particularly when there is competition for health financial resources.

• In LMI countries, service and maintenance must be considered. Service and spare parts are often not readily available and must come from great distances. In the curative treatment of cancer, the impact of equipment 'down-time' may be significant and measurably detrimental.

### New activities launched at ICARO

Two sessions focused on completely new activities which are to be facilitated by the IAEA in the future.

### 1. Quality assurance of international clinical trials

A session was held which reported on the objectives and current status of a working party that is addressing improvements to the implementation of international clinical trials. Harmonization of QA requirements and the streamlining of facility questionnaires were discussed, as were the requirements for databases and digital data submission for improved record collection and analysis. This global working party will meet several times a year to continue the process of analysis and improvement of international clinical trials.

### 2. PACT and manufacturers

A side-meeting with manufacturers of diagnostic and radiotherapy equipment was hosted by IAEA's Programme of Action for Cancer Therapy (PACT) and the Division of Human Health (NAHU). This meeting was convened due to the IAEA's unique and leading role in assisting Member States in the development of cancer therapy, strengthening collaboration with manufacturers in providing equipment that is safe, affordable and technically suitable for developing country conditions. An advisory group was established to continue the process of discussions between the IAEA, manufacturers and users [45].

## Conclusions

Demand for radiotherapy services in LMI countries will increase significantly in the next 20 years. Many Member States are still without or with only very basic radiotherapy facilities. There is a shortage of qualified radiation oncologists, medical physicists, dosimetrists, radiation therapists, nurses, and maintenance engineers in the developing world. Education and training must be provided to meet this demand and training must be ideally adapted to the available equipment and disease profiles.

Since there is competition for health care resources and equipment, technical support has to be consistent with the health system infrastructure of each country to keep radiation treatment affordable, safe and of good quality. In LMI countries, service and maintenance are often not available and must come from afar. This needs to be recognized when purchasing any equipment or technology.

The conference gave delegates of LMI countries an opportunity to assess new technologies relative to their own situations. Many aspects of advances in radiation oncology were covered and evaluated, ranging from the role of basic technology to how to upgrade and adapt departments to advanced technology. The benefits, implications, pitfalls, economics, risks, and practicalities of implementing advances from a variety of viewpoints were discussed.

### Recommendations

• Basic radiation therapy services at a minimum should be made available to all patients with cancer who need them.

• Education and training programmes to enable good quality radiation therapy services need to be developed and job opportunities offered with adequate salary levels to retain staff.

• Advanced technologies in radiation therapy should not be universally adopted until the following conditions are met:

- A need for advanced technology exists (i.e. patients with curative potential)

- Experience with 3D conformal radiation therapy and advanced treatment planning exists before implementation of more advanced technologies

- Adequate imaging services are available

- Studies demonstrate a universal advantage to each aspect of advanced technology, either in improving local control or in reducing toxicity

- Personnel have adequate training in planning, implementation, and QA in advanced technology

- Continuous medical education system is in place.

- An adequate QA/QC programme is in place.

• Clinical studies should be undertaken to demonstrate clinical and cost-effective benefits to the advanced technologies.

• Each country must clearly define which cancer outcomes are expected to be improved by the introduction of advanced technologies.

• New technologies such as IMRT offer theoretical advantage in radiation dose distribution. Presently, there is a paucity of evidence that IMRT can improve tumour-related outcomes, and clinical trials are clearly needed.

• Despite the growing use of protons in various sites including prostate cancer, proton therapy must remain under scrutiny until it has proven itself cost-effective.

## Competing interests

The authors declare that they have no competing interests.

## Authors' contributions

EKS was Scientific Secretary of the ICARO Conference and contributed to drafting and review, KK, GSI and MCJ acted as rapporteurs of the meeting and drafted the initial meeting report, ER, EZ, JW and AM were part of the ICARO Organizing Committee and all contributed to the drafting and review of this article. All authors read and approved the final manuscript.
